# Laboratory Information Management Systems in Oral Pathology: A Comprehensive Review

**DOI:** 10.7759/cureus.60714

**Published:** 2024-05-20

**Authors:** Deeksheetha Prabhu Venkatesh, Karthikeyan Ramalingam, Pratibha Ramani, Deepak Nallaswamy

**Affiliations:** 1 Oral Pathology and Microbiology, Saveetha Dental College and Hospitals, Saveetha Institute of Medical and Technical Sciences, Saveetha University, Chennai, IND; 2 Prosthodontics, Saveetha Dental College and Hospitals, Saveetha Institute of Medical and Technical Sciences, Saveetha University, Chennai, IND

**Keywords:** workflow management, quality control, resource management, resource allocation, optimization techniques, laboratory management, laboratory information, laboratory streamlining, data management, oral pathology lab

## Abstract

Efficiency in oral pathological laboratory processes is paramount for timely and accurate diagnosis. This review explores various strategies and methodologies that help streamline oral pathological laboratory workflows to enhance productivity and reduce turnaround times. Key focus areas include specimen collection, handling, processing, and analysis. Optimization techniques such as automation, digitalization, and standardization are discussed in detail, emphasizing their role in minimizing errors and maximizing throughput. Additionally, the integration of advanced technologies such as artificial intelligence and machine learning is examined for their potential to improve laboratory operations. Moreover, the importance of quality control measures and compliance with regulatory standards is underscored as essential components of any successful laboratory streamlining initiative. By implementing a comprehensive approach that addresses the entire diagnostic pathway, oral pathological laboratories can achieve significant efficiency, ultimately leading to better patient care and outcomes.

## Introduction and background

The primary aim of overseeing a laboratory is to enhance its operations through various tasks. These encompass overseeing diverse aspects such as data and inventory management, personnel supervision, equipment upkeep, and safety protocols [1]. Besides maintaining a secure and organized workspace, the crux of efficient lab management is to bolster productivity and effectiveness. Laboratories play pivotal roles in medical research and treatment by analyzing patient samples for diagnoses and drug development [2]. Competent laboratory management is crucial for carrying out these significant tasks efficiently and with optimal productivity. It is an integral part of scientific research, demanding proper resource allocation, task specification, and employing qualified personnel. Adeptness in conducting experiments and managing research activities is instrumental in ensuring the efficiency of lab management, creating a secure and supportive work environment for all [3].

Institutions and associations involved in research should prioritize lab management to heighten productivity, reduce expenses, and enhance research outcomes. Proficiently managing a lab necessitates judicious utilization of appropriate resources, tools, and supplies to facilitate research endeavors. It has been stressed that resource management is pivotal during research planning and budgeting, potentially enhancing institutional research productivity. Employing resources effectively results in better outcomes, irrespective of their scale. Thus, optimizing resource allocation becomes essential [4]. Competent training and supervision of lab personnel are crucial to ensure efficient and accurate task execution. An effective work environment is pivotal for productivity, necessitating adept management [5].

Continuous training and professional development opportunities are vital for lab workers. Investing in employees’ skills and abilities augments productivity and clinical/research quality. Operating a lab entails more than acquiring equipment; it demands proficient guidance from lab personnel [6]. Proper training enhances technicians’ skills, while supervision ensures adherence to protocols and procedures, preventing declines in quality. Therefore, guidance for lab personnel is imperative for lab managers. Apt human resource management fosters a conducive environment, benefiting researchers/clinicians and research/clinical outcomes alike [7]. It encompasses various aspects, from recruitment and retention to effective task execution.

It is important to keep track of lab experiments to ensure smooth control of the procedures. Quality control protocols aim to confirm that equipment, materials, and procedures meet specified standards and criteria [8]. Quality control helps in minimizing the errors and deviations in the laboratory results. This approach significantly makes the laboratory process more successful and reliable. By putting in place quality control processes, academic institutions can stay one step ahead in detecting any possible problems that could negatively impact their lab reports. Quality control and assurance are vital in lab management, along with conducting regular audits, validation studies, and compliance checks to ensure that the standards in research quality are met [9].

In addition, another important aspect of lab management is ensuring safety and following lab regulations. Lab personnel must maintain a safe and legal standard in their work environment. Safety protocols should not infringe on scientific discovery [10]. It is essential to reduce the possibility of accidents and create a safe environment by following strict rules, giving extensive training, and conducting regular safety checks. It is necessary to keep track of important policy changes that impact the workflow, so as not to inadvertently run into legal and moral predicaments that can hinder developments in the field [3,7].

Lab safety and compliance should always be a high priority in any efficient lab management approach. Hence, effective lab management ensures compliance, optimizes research activities, and fosters a productive environment. Implementing strategies like quality assessment, safety protocols, data management, and strategic planning can enhance effectiveness, potentially elevating performance and reducing costs. These initiatives could contribute significantly to successful research projects and increased scientific knowledge.

## Review

History of laboratory management

The roots of laboratory management extend back to the emergence of modern experimental science during the 17th century when dedicated research spaces started forming. Over time, this evolution has been shaped by scientific progress, technological strides, and the evolving role of research/clinical institutions. This review explores the historical trajectory of lab management, delving into significant moments pivotal to its development [2,3].

The concept of labs as controlled spaces for scientific inquiry dates back to early pioneers like Robert Hooke, Galileo Galilei, and Antonie van Leeuwenhoek. Their systematic experiments in specialized workspaces laid the groundwork for organizing and managing these facilities [1]. The late 19th and early 20th centuries witnessed breakthroughs in chemistry, physics, and biology, leading to specialized research labs in academia and industry. This era formalized the role of lab management, recognizing the necessity for structured approaches to resource organization, research coordination, safety, and compliance [4]. The mid-20th century saw comprehensive frameworks for lab management, emphasizing quality assurance, safety regulations, and standardized procedures in scientific research. Principles from Total Quality Management (TQM) and Good Laboratory Practice (GLP) shaped modern lab management, stressing efficiency, quality control, and compliance were also incorporated in the mid-20th century [4].

From the late 20th century to the early 21st century, digital technologies became integral to labs, spawning tools like laboratory information management system(s) (LIMS) and electronic notebooks. This digital revolution revolutionized lab management, streamlining processes, ensuring data integrity, and fostering collaboration [3]. Lab management has evolved towards a more interdisciplinary approach, recognizing the fusion of management, communication, and innovation in research. Certifications and academic programs can equip researchers and lab managers with the skills needed in the modern world [10].

Lab management’s history is an evolution fueled by scientific discoveries, technology, and societal changes. From its early inception to advanced management principles and digital innovations, lab management continually adapts to meet evolving clinical and research needs. Appreciating this historical journey is crucial to understanding its present state and anticipating future challenges in scientific research management.

Streamlining of laboratory procedures in oral pathology

Enhancing the effectiveness of pathology laboratories is crucial for better patient care. This review explores methods and advancements focused on making these labs more efficient without compromising accuracy. It covers refining processes, using digital tools, and enhancing quality control. Improving how tasks are carried out is a significant part of making oral pathology labs more efficient. Reassessing and redesigning how specimens are managed, processed, analyzed, and reported can notably boost efficiency and reduce wait times. Studies show that adopting Lean and Six Sigma methodologies helps identify and remove inefficiencies, making lab procedures smoother and more effective. Also, employing automation and robotics in handling specimens and analysis decreases errors, increases output, and streamlines workflow [4,5].

The introduction of digital tools like laboratory information management systems, electronic medical records (EMRs), and digital imaging systems has revolutionized lab operations. LIMS aids in tracking specimens and results while facilitating communication among lab staff, clinicians, and patients. EMRs enable quick access to patient data and results, expediting diagnosis. Additionally, digital imaging tools like whole-slide imaging enhance how pathologists review tissue samples, improving accuracy and collaboration both within and outside the institution [10].

Maintaining quality is crucial in oral pathology labs to ensure accurate results. Accreditation programs provide a structure for implementing quality assurance and proficiency testing [11]. Also, adhering to standard procedures, quality control measures and regular audits uphold high standards in testing. The advancements in molecular diagnostics and personalized medicine have impacted lab processes [12]. Continuous innovation in genomics, proteomics, and molecular pathology requires labs to adapt their processes and infrastructure. Implementing these advanced techniques in routine workflows demands careful validation, interpretation, and reporting of tests, alongside specialized expertise and equipment [13].

**Figure 1:**
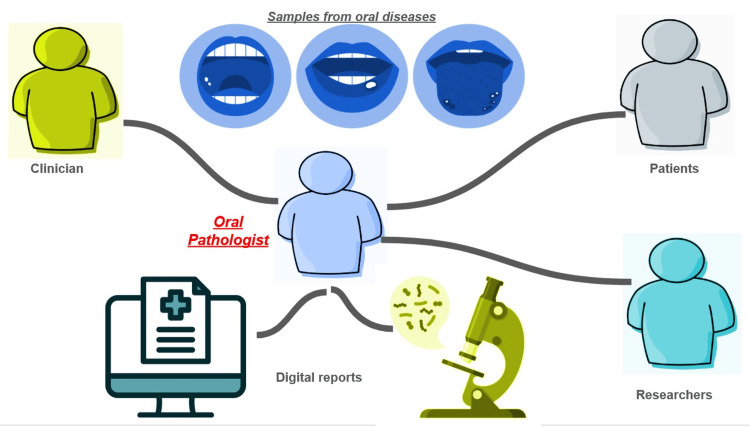
Interactions of an oral pathologist Picture credit: Dr. Karthikeyan Ramalingam

Ensuring uniformity and consistency in lab practices is vital in optimizing oral pathology labs, especially in settings with multiple locations [14]. Employing standardized procedures, test methods, and report formats across various lab sites can enhance result uniformity, comparability, and resource efficiency. This uniform approach is crucial, particularly within interconnected healthcare networks or when serving diverse patient groups (Figure 1) [15].

On-the-spot testing in pathology labs has streamlined urgent or critical care diagnostics [16]. Devices for immediate blood analysis like blood gases, glucose, and coagulation parameters expedite decision-making and improve patient outcomes. However, integrating point-of-care devices into lab workflows demands stringent quality control, supervision, and integration with primary lab systems [17].

Integrating eco-friendly practices into lab management aligns with the healthcare industry’s focus on sustainability. Initiatives like using energy-efficient equipment, reducing waste, and adopting environmentally conscious lab practices can save costs, comply with national and international regulations, and lessen environmental impact. Infusing sustainability into lab management showcases a commitment to responsible healthcare practices [18]. Additionally, the emergence of remote pathology services has streamlined lab operations by enabling distant access to expert consultations and collaborative case assessments [19]. Platforms for telepathology facilitate the digital sharing and review of specimen images and diagnostic data, fostering efficient collaboration between oral pathologists, clinicians, and researchers. This approach is valuable in providing specialized oral pathology expertise to underserved or remote areas [20].

The utilization of artificial intelligence (AI) has become an encouraging approach to enhance the efficiency of oral pathology laboratories. AI-driven algorithms and machine learning applications are being designed to aid in activities like slide screening, pattern identification, and providing support for diagnostic decision-making. AI could improve the effectiveness of oral pathology labs, minimize diagnostic inaccuracies, and elevate the overall precision of test analyses. However, the incorporation of AI into lab processes must undergo thorough validation and integration to guarantee its suitability for clinical use and compliance with regulatory standards [21].

An inclusive strategy to streamline pathology labs involves incorporating various technologies, maintaining standardization, embracing on-the-spot testing, adopting sustainable practices, and utilizing telepathology solutions [22]. These approaches can enhance lab diagnostic capabilities, elevate patient care outcomes, and contribute significantly to diagnostic medicine advancement. Making pathology labs more efficient involves refining workflows, adopting digital solutions, ensuring quality control, and adapting to new diagnostic technologies [23]. Implementing strategies to enhance efficiency, leveraging digital tools, maintaining quality, and embracing new diagnostic methods can improve lab capabilities, benefiting patient care and contributing to precision medicine advancements [24].

Laboratory information management system

In today's world of scientific research, medical advancements, and industrial discoveries, efficiently managing laboratory data is critical for success in various fields [25]. LIMS have been concurrently employed in various modern laboratories to provide researchers, lab directors, and corporate executives with the necessary aids for navigation of issues with data management [20]. The duties of laboratory management are to manage the steps of sample tracking, result interpretation, and report creation. If information is not effectively managed and organized, it can quickly become overwhelming and could have a hazardous impact on patient outcomes [22].

LIMS aims to gather, store, and manage data from various laboratory activities. LIMS aids pathologists and lab personnel in efficiently tracking samples from collection to reporting. Automation of lab procedures using LIMS can reduce errors caused by manual methods and save valuable time [23].

LIMS From Theory to Practice

LIMS used in laboratories employs computers and digital software to manage and store information. It involves tracking of the sample from its receipt to the final step of reporting and analysis [18]. Although the concept of LIMS was put forth in the early 1960s, it was brought to fruition only in the late 1980s.

Three types of LIMS have been described. Type 1 is an operational LIMS consisting of an automated process that replaces the manual processes in the analytical process carried out in the laboratory. Type 2 is a logistical type of LIMS, which improves the effectiveness of the laboratory process by providing pathologists and technicians with the required information to carry out their duties. Type 3 is a strategic type of LIMS that integrates information and applications from different functional areas, and operations can be reshaped to improve the competency of the laboratory [17].

Impact of LIMS in a Laboratory Setting

LIMS can standardize the various procedures and processes that are carried out in the laboratory while reducing ambiguities and human error. It accelerates the rate at which information such as results are delivered while reducing the time taken between sample collection and report generation by stimulating the need for demand and increasing productivity. It makes the laboratory an information-based organization and transforms its self-sustaining capabilities [16,19].

Its disadvantages are sample rejection can occur due to technical errors, and implementation of LIMS in a lab can be arduous [19].

LIMS in India

The Bureau of Indian Standards (BIS) comes under the National Standards Body of India [26]. Laboratories can submit relevant data about lab catalogs, tests performed, and manpower details for BIS approval. It should include quality formats, inter-lab comparison, efficiency testing, internal audit, management review, and scope managed through LIMS. A PubMed search revealed very few articles for the terms ("laboratory information management system"[All Fields]) AND ("india"[All Fields]). LIMS has been implicated in microbiology, bioinformatics and agriculture [27-29]. There were no studies on oral pathology in PubMed.

LIMS protocol followed at our institution

At our institution (Saveetha Dental College and Hospitals), like LIMS, Dental Information Archival System (DIAS) is used and is an indigenous software developed by Dr. Deepak Nallaswamy. It integrates all patient data under one roof and contains all information from the patient’s entry registration to the patient’s entire course in the hospital.

**Figure 2:**
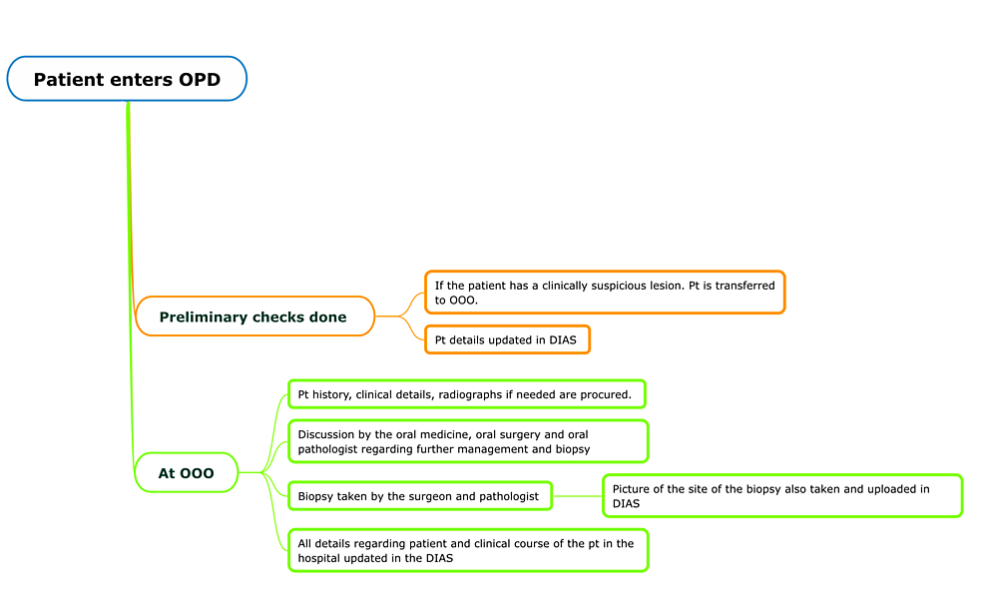
Clinical course of a patient and digital workflow in the Dental Information Archival System (DIAS) Pt, patient; OOO, Department of Oral Medicine, Oral Surgery, and Oral Pathology Picture credits: Dr. Deeksheetha Prabhu, Dr. Karthikeyan Ramalingam

The preliminary checks of a patient with clinically suspicious lesions are done at the Department of Oral Medicine, Oral Surgery, and Oral Pathology (OOO), and the details are uploaded onto the DIAS. At the OOO, patient history, clinical details, and radiographs are taken and after a further discussion between the respective physicians, a biopsy is taken from a representative site. The biopsy is taken by the oral surgeon after consultation with the pathologist. Pictures of the biopsy site are also taken and uploaded onto the DIAS. all details regarding the patient and clinical course of the patient are updated in the DIAS (Figure 2).

**Figure 3:**
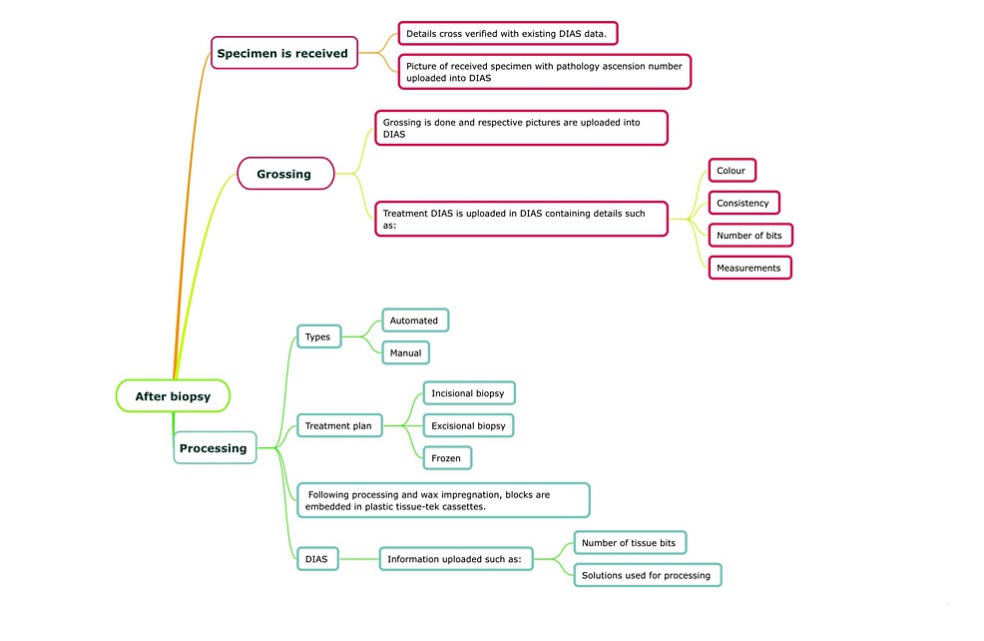
Digital workflow in the Dental Information Archival System (DIAS), after biopsy Picture credits: Dr. Deeksheetha Prabhu, Dr. Karthikeyan Ramalingam

After the biopsy, the specimen is placed in 10% neutral buffered formalin, and a digital biopsy request is sent to the oral pathology department containing the details of the biopsy procedure and the clinical details. The specimen is received in the oral pathology department. Grossing is carried out and the respective pictures are uploaded onto the DIAS (Figure 3).

**Figure 4:**
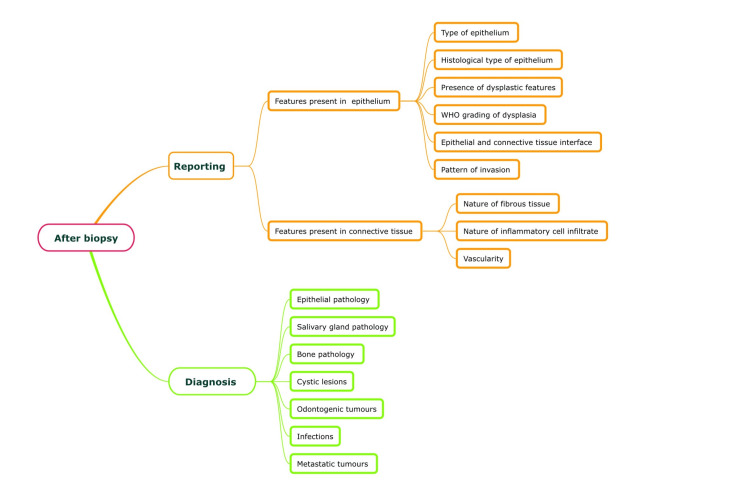
Information flow for the biopsy specimen diagnosis in the Dental Information Archival System (DIAS) Picture credits: Dr. Deeksheetha Prabhu, Dr. Karthikeyan Ramalingam

Following grossing, information such as several tissue bits and solutions used for tissue processing are uploaded onto the DIAS. After embedding the processed tissues, sectioning and staining are carried out, and the slides are stained with hematoxylin and eosin. Reporting is carried out and various features of the epithelium and connective tissue are mentioned in the DIAS. The diagnosis for the biopsy specimen is also uploaded onto the DIAS and is segregated into the origin of the lesion. This biopsy report is instantly available for the clinicians (Figure 4).

## Conclusions

Effective management of an oral pathology lab is vital to ensure precise diagnostic outcomes, uphold operational efficiency, and improve patient care. By adopting efficient procedures, utilizing cutting-edge technologies like LIMS, and emphasizing quality control, labs can boost productivity, decrease turnaround times, and reduce errors. As pathology labs adapt to technological progress and evolving healthcare demands, strategic management approaches will be key in achieving success and meeting the needs of both patients and healthcare professionals. In our institution, DIAS is employed for all patients and it creates an ease of communication between the clinicians and oral pathologists while providing readily available clinical information for the pathologist for ease of diagnosis. Likewise, it also provides readily available results for clinicians. Moreover, physicians have constant access to all patient data for subsequent treatment and management purposes, which can improve treatment efficacy while maintaining efficiency.
